# Circulating plasmablasts/plasma cells: a potential biomarker for IgG4-related disease

**DOI:** 10.1186/s13075-017-1231-2

**Published:** 2017-02-10

**Authors:** Wei Lin, Panpan Zhang, Hua Chen, Yu Chen, Hongxian Yang, Wenjie Zheng, Xuan Zhang, Fengxiao Zhang, Wen Zhang, Peter E. Lipsky

**Affiliations:** 1 Department of Rheumatology, Peking Union Medical College Hospital, Chinese Academy of Medical Sciences, Key Laboratory of Rheumatology and Clinical Immunology, Ministry of Education, Beijing, 100730 China; 2 Department of Rheumatology, Hebei General Hospital, Shijiazhuang, China; 3 National Institutes of Health, Bethesda, MD USA

**Keywords:** IgG4-RD, Biomarker, Autoimmunity, CD19^+^CD24^−^CD38^hi^ plasmablast/plasma cell

## Abstract

**Background:**

Immunoglobulin G4 (IgG4)-related disease (IgG4-RD) is a multisystem fibroinflammatory disease. We previously reported that a circulating cell population expressing CD19^+^CD24^−^CD38^hi^ was increased in patients with IgG4-RD. In this study, we aimed to document that this cell population represented circulating plasmablasts/plasma cells, to identify the detailed phenotype and gene expression profile of these IgG4-secreting plasmablasts/plasma cells, and to determine whether this B-cell lineage subset could be a biomarker in IgG4-related disease (IgG4-RD).

**Methods:**

A total of 42 untreated patients with IgG4-RD were evaluated. Peripheral B-cell subsets, including CD19^+^CD24^−^CD38^hi^ plasmablasts/plasma cells, CD19^+^CD24^+^CD38^−^ memory B cells, CD19^+^CD24^int^CD38^int^ naïve B cells, and CD19^+^CD24^hi^CD38^hi^ regulatory B cells, were assessed and sorted by flow cytometry. Microarray analysis was used to measure gene expression of circulating B-cell lineage subsets. Further characterization of CD19^+^CD24^−^CD38^hi^ plasmablasts/plasma cells was carried out by evaluating additional surface markers, including CD27, CD95, and human leukocyte antigen (HLA)-DR, by flow cytometric assay. In addition, various B-cell lineage subsets were cultured in vitro and IgG4 concentrations were measured by cytometric bead array.

**Results:**

In untreated patients with IgG4-RD, the peripheral CD19^+^CD24^−^CD38^hi^ plasmablast/plasma cell subset was increased and positively correlated with serum IgG4 levels, the number of involved organs, and the IgG4-related Disease Responder Index. It decreased after treatment with glucocorticoids. Characterization of the plasmablast/plasma cell population by gene expression profiling documented a typical plasmablast/plasma cell signature with higher expression of X-box binding protein 1 and IFN regulatory factor 4, but lower expression of paired box gene 5 and B-cell lymphoma 6 protein. In addition, CD27, CD95, and HLA-DR were highly expressed on CD19^+^CD24^−^CD38^hi^ plasmablasts/plasma cells from patients with IgG4-RD. Furthermore, CD19^+^CD24^−^CD38^hi^ plasmablasts/plasma cells secreted more IgG4 than other B-cell populations.

**Conclusions:**

Circulating CD19^+^CD24^−^CD38^hi^ plasmablasts/plasma cells are elevated in active IgG4-RD and decreased after glucocorticoid treatment. This IgG4-secreting plasmablast/plasma cell population might be a potentially useful biomarker for diagnosis and assessing response to treatment.

## Background

Immunoglobulin G4 (IgG4)-related disease (IgG4-RD) is a newly recognized fibroinflammatory disease characterized by infiltration of IgG4^+^ plasma cells together with distinct storiform fibrosis in involved lesions and significantly increased serum IgG4 levels [1–3]. There are various clinical manifestations in patients with IgG4-RD, including Mikulicz’s disease, autoimmune pancreatitis, IgG4-related retroperitoneal fibrosis, pulmonary involvement, kidney disease, sclerosing cholangitis, sinusitis, sclerosing thyroiditis, and others [4–12].

IgG4-RD is characterized by a number of abnormalities in the differentiation of cells of the B-cell lineage, including increased serum levels of IgG, IgG4, and often IgE; infiltration of affected tissues by IgG4-secreting plasma; and the presence of increased frequencies of circulating plasma cells/plasmablasts [13–15]. B cells and plasma cells may play important roles in the development of this disease [16]. Our previous studies revealed that, compared with healthy control subjects and patients with primary Sjögren’s syndrome, patients with IgG4-RD expressed an increased circulating population of CD19^+^CD24^−^CD38^hi^ cells that appeared to be circulating plasmablasts/plasma cells and correlated positively with serum IgG4 levels [17]. The full characterization of this circulating population was not carried out, but its appearance and correlation with serum IgG4 suggested that it might be an important biomarker of IgG4-RD. To begin to address these questions, we initially characterized the circulating plasmablasts/plasma cells in IgG4-RD in greater detail.

## Methods

### Patients

Forty-two untreated patients with IgG4-RD fulfilling the 2011 comprehensive IgG4-RD diagnostic criteria were enrolled in this study. The diagnosis of IgG4-RD was based on the following three manifestations: (1) clinical examination showing characteristic diffuse/localized swelling or masses in single or multiple organs; (2) hematological examination showing elevated serum IgG4 concentration (>135 mg/dl); and (3) histopathologic examination showing (a) marked lymphocyte and plasma cell infiltration and fibrosis or (b) infiltration of IgG4^+^ plasma cells (ratio of IgG4^+^/IgG^+^ cells >40% and >10% IgG4^+^ plasma cells per high-power field). Patients with cancer or lymphoma and other autoimmune diseases were excluded.

### Clinical data and inflammatory parameters

Clinical data, including age, gender, disease duration, and manifestations, were obtained for all patients. Laboratory findings were recorded, including erythrocyte sedimentation rate (ESR); C-reactive protein (CRP); and serum immunoglobulin IgG, IgA, IgM, and IgG subsets. IgG4-related Disease Responder Index (IgG4-RD RI) was calculated for each patient [18].

### Flow cytometric analysis and separation of B-cell subpopulations

Peripheral blood mononuclear cells (PBMCs) from patients with IgG4-RD were separated by Ficoll gradient centrifugation. Different B-cell populations were stained with phycoerythrin (PE)-cyanine 7 (Cy7)-anti-CD19, fluorescein isothiocyanate-anti-CD24, allophycocyanin-anti-CD38, peridinin chlorophyll protein-CY5.5-anti-CD27, PE-anti-CD40, PE-anti-CD80, PE-anti-CD86, PE-anti-B-cell activating factor receptor (BAFF-R), PE-anti-transmembrane activator CAML (calcium modulator and cyclophilin ligand) interactor (TACI), PE-anti-CD95, PE-anti-CD138, PE-anti-interleukin-6 receptor (IL-6R), PE-anti-IgD, and PE-anti-CD59 monoclonal antibodies (mAbs) (BD Biosciences, San Jose, CA, USA), as well as PE-anti-B-cell maturation antigen (BCMA) mAb (BioLegend, San Diego, CA, USA), or isotype-matched controls. After incubation for 30 minutes at 4 °C, the cells were washed and resuspended in fluorescence-activated cell sorting staining buffer (BD Biosciences). All experiments were analyzed by gating on lymphocytes according to forward scatter/side scatter; dead or dying cells or granulocytes were excluded. B-cell subsets were gated on CD19 and then gated on CD24 and CD38. Flow cytometric analysis was performed immediately after sample preparation. All samples were analyzed using a BD FACSAria II system (BD Biosciences), and data were analyzed by FlowJo version 7.6.4 software (FlowJo, Ashland, OR, USA).

PBMCs were stained with CD19, CD24, and CD38 antibodies and then sorted with a MoFlo high-performance cell sorter (Beckman Coulter Life Sciences, Indianapolis, IN, USA). Different B-cell subsets were separated, including CD19^+^CD24^+^CD38^−^ memory B cells, CD19^+^CD24^int^CD38^int^ naïve B cells, CD19^+^CD24^hi^CD38^hi^ regulatory B cells (Bregs), and CD19^+^CD24^−^CD38^hi^ plasmablasts/plasma cells. Sorted B-cell populations with purity greater than 95% were used for in vitro culture and RNA extraction.

### In vitro cell culture

Purified B-cell populations from patients with IgG4-RD were resuspended in RPMI 1640 medium supplemented with 10% fetal calf serum and antibiotics (penicillin 100 IU/ml, streptomycin 100 μg/ml; Life Technologies, Carlsbad, CA, USA) in 96-well U-bottomed plates (Nunc, Langenselbold, Germany) in a humidified atmosphere of 5% CO_2_ at 37 °C with 1 × 10^5^ cells in each well. For each group, 100 ng/ml recombinant human CD40L (Abcam, Cambridge, MA, USA) and 0.1 μg/ml cytosine phosphate guanosine oligodeoxynucleotide 2006 (CpG ODN 2006; InvivoGen, San Diego, CA, USA) was added at the beginning. After 7 days of culture, the levels of Ig secretion in collected supernatants were tested.

### Cytometric bead array analysis

Culture supernatant samples from different B-cell subsets were collected and stored at −80 °C until used. Cytometric bead array (CBA) analysis for IgG, IgA, IgM, IgG4, and IgE in supernatants was performed according to the manufacturer’s instructions (BD Biosciences). Data were analyzed using CBA analysis software obtained from BD Biosciences. The concentrations of IgG, IgA, IgM, IgG4, and IgE in supernatants were determined by reference to a standard curve.

### Microarray analysis of gene expression

Sorted B-cell subpopulations were placed in TRIzol reagent (Life Technologies) for RNA extraction following the manufacturer’s instructions. Isolated RNA was further purified with the RNeasy Mini Kit (Qiagen, Valencia, CA, USA) and processed for microarray analysis using the standard Affymetrix protocols (www.affymetrix.com; Affymetrix, Santa Clara, CA, USA). Briefly, 1–10 μg of RNA was reverse-transcribed into complementary DNA (cDNA) (Life Technologies). The template cDNA was purified for amplification and in vitro transcription to cRNA using the BioArray™ HighYield™ RNA Transcript Labeling Kit (T7) (Enzo Life Sciences, Inc., Farmingdale, NY, USA). cRNA was biotin-labeled, purified, and hybridized to Human Genome U133A GeneChips® (Affymetrix). GeneChips® were scanned on a high-resolution scanner using GCOS version 1.2 software (Affymetrix). Data analysis was conducted after standard Affymetrix algorithm analysis (MAS5).

### Statistical methods

Statistical analyses were performed using IBM SPSS Statistics version 19.0 software (IBM, Armonk, NY, USA) and Prism software version 5.0 (GraphPad Software, La Jolla, CA, USA). A *P* value <0.05 was considered significantly different. Data are reported as mean ± SD. Normal distribution data between two groups were analyzed using independent-samples *t* tests or paired-samples *t* tests, and one-way analysis of variance (ANOVA) was used to compare groups. The relationships between CD19^+^CD24^−^CD38^hi^ plasmablasts/plasma cells and clinical features were analyzed by Pearson’s rank correlation test, and a *P* value <0.05 was considered significant. The level of gene expression was standardized by robust multiarray average and detection above background. One-way ANOVA was used to test different levels of gene expression. The Benjamini-Hochberg method was employed to determine the false discovery rate after multiple hypothesis testing. A false discovery rate <0.3 was used.

## Results

### Characteristics of patients with IgG4-RD

All 42 patients were newly diagnosed, untreated patients with IgG4-RD. Their demographic features as well as clinical and laboratory manifestations are listed in Table 1. Their average age was 55 (41.5–60) years old, and the male-to-female ratio was 2.23:1. Thirty-two (76.2%) of the patients were characterized as definite IgG4-RD, 1 (2.4%) was classified as probable IgG4-RD, and 9 (21.4%) were categorized as possible IgG4-RD. The majority of patients had multiple organ involvement. Forty-one (97.6%) patients had elevated serum IgG4.

**Table 1:** Characteristics of 42 patients with immunoglobulin G4-related disease

Characteristics	Data
Age at evaluation, years, median (IQR)	55 (41.5–60)
Male/female ratio	2.23:1
Baseline responder index, median (IQR)	14.83 (5.99)
Diagnosis, n (%)	
Definite	32 (76.2)
Probable	1 (2.4)
Possible	9 (21.4)
Disease duration, months, median (IQR)	12 (5.75–36)
Number of organs involved, mean (SD)	4.33 (1.79)
Organs involved, n (%)	
1	1 (2.4)
2	3 (7.1)
3 or more	38 (90.5)
Lymph nodes involved, n (%)	37 (88.1)
Salivary glands involved, n (%)	24 (57.1)
Lacrimal glands involved, n (%)	24 (57.1)
Pancreas involved, n (%)	13 (31.0)
Lung involved, n (%)	13 (31.0)
Prostate involved of male, n (%)	12 (41.4)
Paranasal sinus involved, n (%)	11 (26.2)
Retroperitoneal organs involved, n (%)	11 (26.2)
Bile duct involved, n (%)	8 (19.0)
Kidney involved, n (%)	6 (14.3)
Skin involved, n (%)	4 (9.5)
History of allergy, n (%)	29 (69.0)
Elevated serum IgG4 concentration at baseline, n (%)	41 (97.6)
Elevated IgE, n (%)	26/29 (89.7)
Eosinophilia, n (%)	16 (38.1)

*Ig* Immunoglobulin

### Correlations of CD19^+^CD24^−^CD38^hi^ plasmablasts/plasma cells with clinical and laboratory parameters in patients with IgG4-RD

**Fig. 1:**
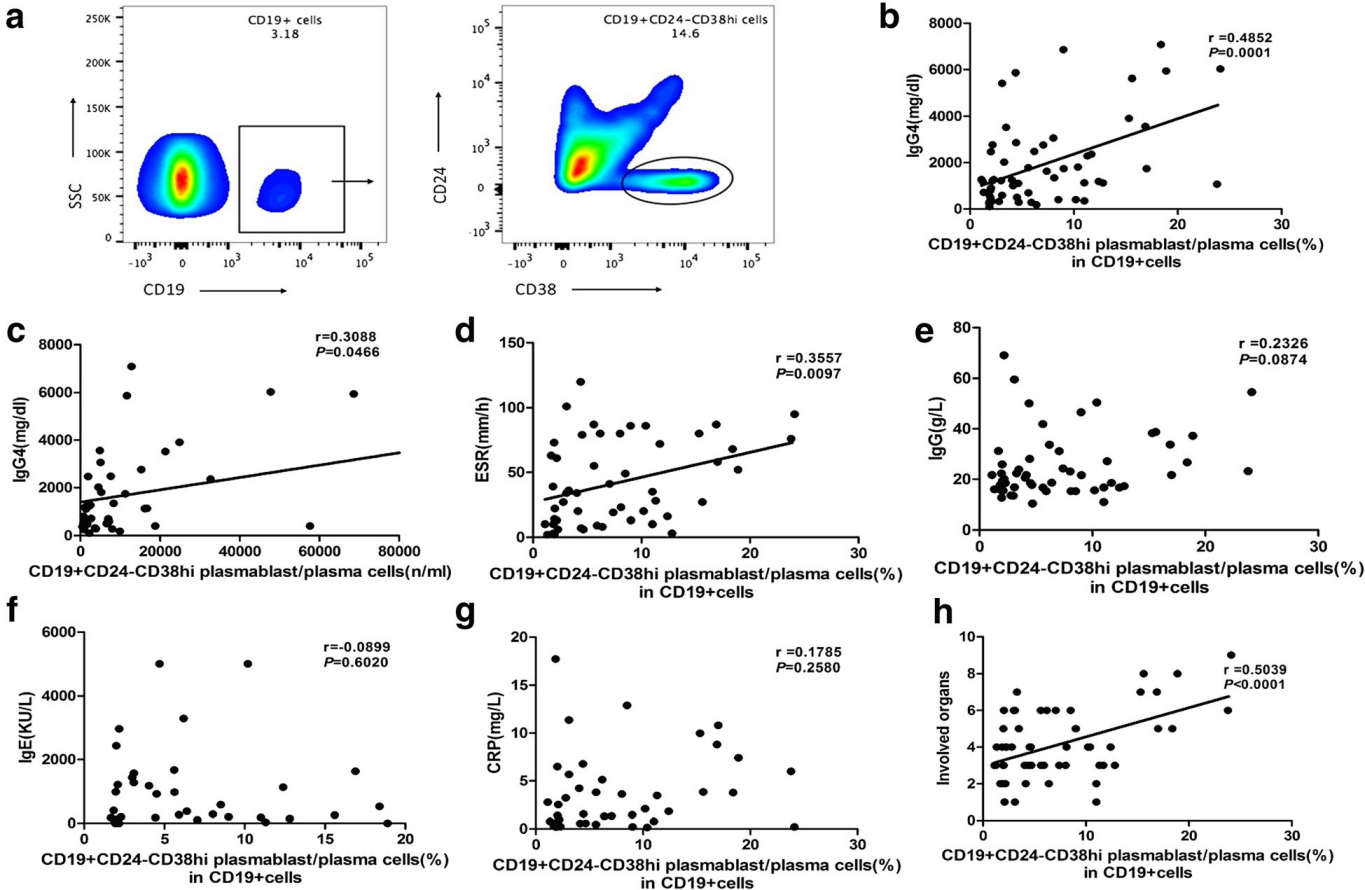
The correlations of CD19^+^CD24^−^CD38^hi^ plasmablasts/plasma cells with clinical and laboratory parameters. **a** Gating strategy of CD19^+^CD24^−^CD38^hi^ plasmablasts/plasma cells. Correlations of the ratio of CD19^+^CD24^−^CD38^hi^ plasmablasts/plasma cells and serum (**b**) immunoglobulin G4 (IgG4), (**d**) erythrocyte sedimentation rate (ESR), (**e**) IgG, (**f**) IgE, (**g**) C-reactive protein (CRP), and (**h**) involved organs are shown. **c** Correlation of the absolute number of CD19^+^CD24^−^CD38^hi^ plasmablasts/plasma cells and serum IgG4. *SSC* Side scatter

Our previous studies revealed that CD19^+^CD24^−^CD38^hi^ plasmablasts/plasma cells were significantly increased in the peripheral blood of patients with IgG4-RD (6.99 ± 6.24%), higher than that in patients with Sjögren’s syndrome (2.39 ± 2.64%, *P* < 0.001) and healthy control subjects (2.16 ± 1.65%, *P* < 0.001) [17]. The plot with the gating strategy for identifying the different B-cell subpopulations is shown in Fig. 1a. In this study, we further analyzed the association of these B-lineage populations with clinical and laboratory parameters of IgG4-RD. Figure 1b–h illustrates the correlations between CD19^+^CD24^−^CD38^hi^ plasmablasts/plasma cells and clinical/laboratory parameters and shows that the ratio of CD19^+^CD24^−^CD38^hi^ plasmablasts/plasma cells/CD19^+^ B cells correlated positively with serum IgG4 (*R* = 0.4852, *P* = 0.0001), the numbers of involved organs (*R* = 0.5039, *P* < 0.0001), and ESR (*R* = 0.3557, *P* = 0.0097), but that it had no correlation with serum IgG (*P* = 0.0874), serum IgE (*P* = 0.6020), or CRP (*P* = 0.2580). In addition, the absolute number of CD19^+^CD24^−^CD38^hi^ cells in patients with IgG4-RD was markedly elevated at 7100/ml (2711–16,297/ml), compared with 1475/ml (1021–2792/ml) in healthy subjects (*P* < 0.001). Importantly, the absolute number of CD19^+^CD24^−^CD38^hi^ plasmablasts/plasma cells significantly correlated with serum IgG4 as well (*R* = 0.3088, *P* = 0.0466) (Fig. 1c).

### Differences in gene expression profiling among CD19^+^C24^−^CD38^hi^ plasmablasts/plasma cells, naïve B cells, memory B cells, and regulatory B cells

**Fig. 2:**
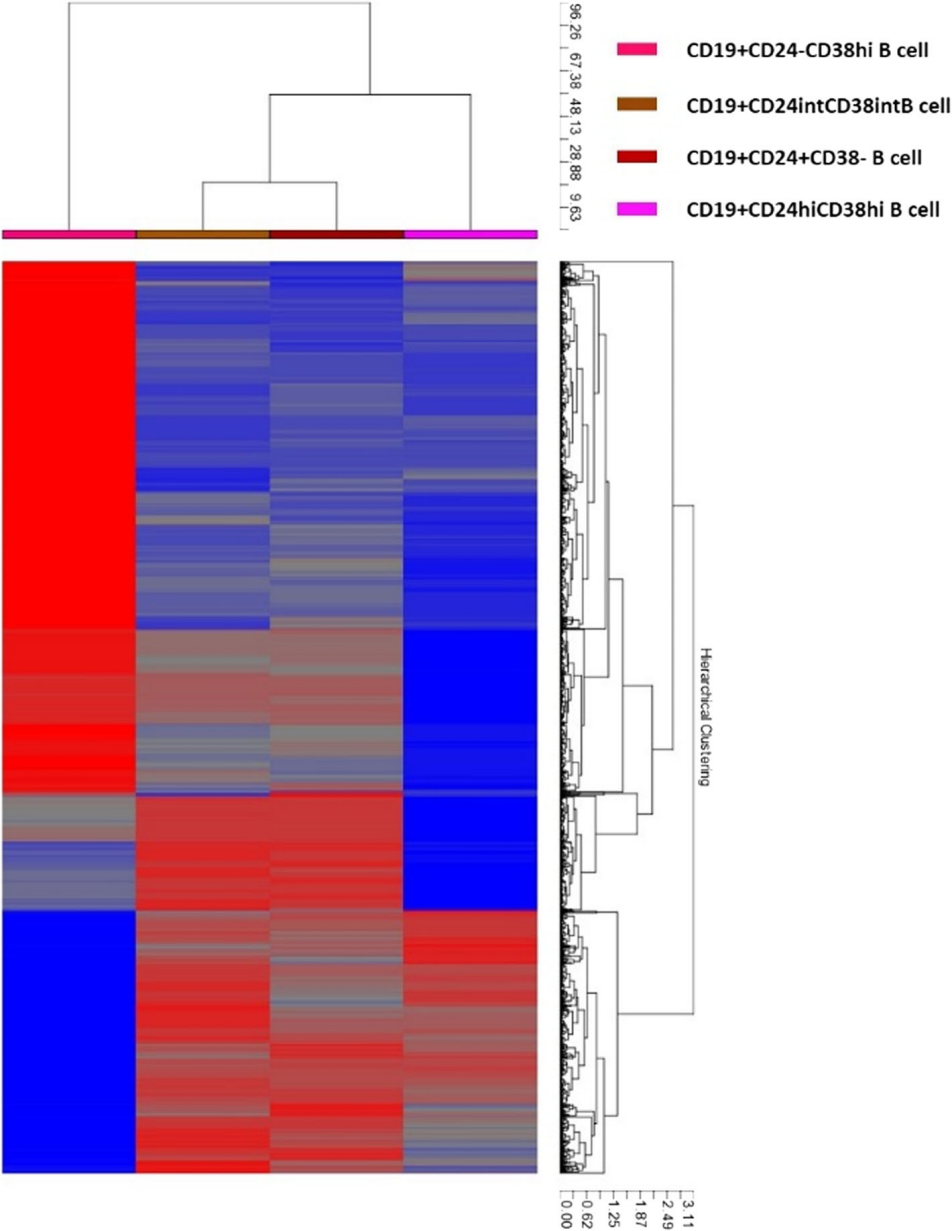
Gene expression of CD19^+^CD24^−^CD38^hi^ plasmablasts/plasma cells, regulatory B cells (Bregs), memory B cells, and naïve B cells in patients with immunoglobulin G4-related disease (IgG4-RD). B-cell subsets were sorted from the peripheral blood of patients with IgG4-RD, and total RNA was extracted for microarray analysis. The differences among CD19^+^CD24^−^CD38^hi^ plasmablasts/plasma cells and other B-cell subsets are shown. Columns from left to right represent CD19^+^CD24^−^CD38^hi^ plasmablasts/plasma cells, CD19^+^CD24^int^CD38^int^ naïve B cells, CD19^+^CD24^+^CD38^−^ memory B cells, and CD19^+^CD24^hi^CD38^hi^ Bregs, respectively. *Red* represents overexpressed genes, and *blue* represents underexpressed genes

To further analyze the characteristics of CD19^+^CD24^−^CD38^hi^ plasmablasts/plasma cells, we collected PBMCs from 15 newly diagnosed patients with IgG4-RD, and the following B cell subsets were sorted by flow cytometry: Bregs (CD19^+^CD24^hi^CD38^hi^), memory B cells (CD19^+^CD24^+^CD38^−^), naïve B cells (CD19^+^CD24^int^CD38^int^), and plasmablasts/plasma cells (CD19^+^CD24^−^CD38^hi^). Because the numbers of each B-cell subset were small, the subsets of B cells from individual patients were pooled. Total RNA was extracted for microarray analysis. Hierarchical clustering visualized by heat mapping indicated significant differences in gene expression by the four B-cell subsets (Fig. 2). Importantly, CD19^+^CD24^−^CD38^hi^ plasmablasts/plasma cells were the most different from the other populations. Among genes that were differentially upregulated in CD19^+^CD24^−^CD38^hi^ plasmablasts/plasma cells compared with naïve B cells and memory B cells, we found those that are known to alter the B-cell program and commit a B cell to plasma cell differentiation, such as interferon regulatory factor 4 (*IRF4*), PR domain containing 1 (*PRDM1*), and X-box binding protein 1 (*XBP1*). Ig heavy and light chains as well as J chain were also significantly upregulated, in addition to those previously reported to be upregulated in plasma cell genes, such as *VDR*, *CAV1, SDC1, SSR4, ERP70, PPIB, ITGA6, CD38,* and others listed in Tables 2 and 3 [19–26]. Both *IGK* and *IGL* genes were upregulated, implying the polyclonal nature of the plasma cell expansion. Genes involved in plasma cell homing to tissue niches such as *SDC1* were also upregulated, although* CXCR4*, which is also involved in homing of plasma cells to niches, was downregulated. Finally, tumor necrosis factor receptor superfamily member 17 (*TNFRSF-17*), which encodes *BCMA*, a BAFF-R involved in plasma cell survival, was greatly upregulated in CD19^+^CD24^−^CD38^hi^ plasmablasts/plasma cells. As expected, BCR complex, *CD19, CD20, CIITA*, human leukocyte antigen *(HLA)-DRA*, and *HLA-DRB1* gene expression was downregulated in CD19^+^CD24^−^CD38^hi^ plasmablasts/plasma cells. Likewise, the genes characteristic of earlier stages of B-cell development or differentiation, such as *SPIB, BCL6, BLK, FYN, BCL11A, CD37, CD1C, VAV3, CCR6,* and *CD22*, were downregulated significantly.

**Table 2:** Genes upregulated in CD19^+^CD24^−^CD38^hi^ plasmablasts/plasma cells of patients with immunoglobulin G4-related disease

Gene symbol	Fold change	Function
*XBP1*	17.2451	Transcription factor
*ESR1*	3.50117	Transcription factor
*PRDM1*	6.7131	Transcription factor
*IRF4*	3.88718	Transcription factor
*TLR8*	4.81882	Cell surface, intracellular signaling
*SDC1*	2.99145	Cell surface, intracellular signaling
*CD59*	12.8752	Cell surface, complexes with integrin
*CD38*	32.2263	Cell surface, intracellular signaling
*IL6R*	6.89689	Cell surface
*PPIB*	6.5266	Chaperone, unfolded protein response
*HSPA13*	7.91297	Chaperone, unfolded protein response
*DNAJC1*	3.01175	Chaperone, unfolded protein response
*DNAJB9*	2.97997	Chaperone, unfolded protein response
*SSR3*	6.50177	Protein assembly and trafficking
*SSR4*	5.70331	Protein assembly and trafficking
*TNFRSF17*	32.2516	Inhibitor of apoptosis
*MAN1A1*	5.35643	Posttranscriptional modification
*VDR*	16.5348	Transcriptional regulatory activity
*ITGA6*	2.21157	Integrin
*IGHG*	6.5159	Immunoglobulin heavy chain
*IGH@*	6.4298	Immunoglobulin heavy chain
*IGHA1*	4.101	Immunoglobulin heavy chain
*IGLC1*	7.9258	Immunoglobulin light chain
*IGLJ3*	3.789	Immunoglobulin light chain
*IGLL5*	9.8509	Immunoglobulin light chain
*IGLV1-44*	8.2352	Immunoglobulin light chain
*IGK@*	6.8981	Immunoglobulin kappa gene
*IGKC*	7.1539	Immunoglobulin kappa gene
*CD27*	4.65501	Apoptosis, signal transduction
*CAV1*	27.0908	Intracellular
*IGF1*	3.64001	Signal transduction
*LTK*	3.27963	Tyrosine kinase, intracellular signaling
*GADD45A*	4.13495	Cell cycle
*CD44*	2.23611	Cellular migration, cytokine receptor activity
*MKI67*	5.39044	Cell proliferation
*CCR10*	2.16384	Chemokine receptor

**Table 3:** Genes downregulated in CD19^+^CD24^−^CD38^hi^ plasmablasts/plasma cells of patients with immunoglobulin G4-related disease

Gene symbol	Fold change	Function
*TRF8*	–7.34056	Transcription factor
*SPIB*	–4.91502	Transcription factor
*PAX5*	–7.43584	Transcription factor
*FOXO1*	–3.15445	Transcription factor, anti-apoptosis
*STAT6*	–3.06941	Transcription factor, signal transduction
*CD72*	–17.9585	Cell surface, intracellular signaling
*CD37*	–9.23361	Cell surface, intracellular signaling
*CD40*	–4.37638	Cell surface, signal transduction
*CD24*	–22.3673	Cell surface, intracellular signaling
*CD1C*	–3.49943	Cell surface
*CD19*	–2.03392	Cell surface, signal transduction
*SELL*	–2.59485	Cell surface, adhesion receptors
*CD22*	–8.40913	Cell surface, intracellular signaling
*CXCR4*	–5.3050	Chemokine receptor
*CXCR5*	–11.9723	Chemokine receptor
*CCR6*	–20.5228	Chemokine receptor
*CCR7*	–9.86589	Chemokine receptor
*TNFSF12* *TNFSF12-TNFSF13*	–2.22538	Signal transduction
*VAV3*	–10.284	Signal transduction
*IL6*	–5.19	Cytokine
*IL7*	–9.61703	Cytokine
*HLA-DRA*	–3.2849	Peptide antigen binding
*BCR*	–7.43584	Kinase activity, protein binding
*HLA-DRB1*	–4.292155	Peptide antigen binding
*FYN*	–2.82807	Tyrosine kinase
*BLK*	–6.10205	Tyrosine kinase
*BCL11A*	–14.6221	Transcriptional regulation activity
*BACH2*	–14.6267	Transcriptional regulation activity
*ID3*	–2.80866	Transcriptional regulation activity
*CIITA*	–4.67153	HIA class II complex, transcription coactivator
*IGF1R*	–5.53806	Inhibitor of apoptosis
*ITGB2*	–2.37331	Integrin

### B-cell differentiation-related transcription factor genes in CD19^+^CD24^−^CD38^hi^ plasmablasts/plasma cells in patients with IgG4-RD

**Fig. 3:**
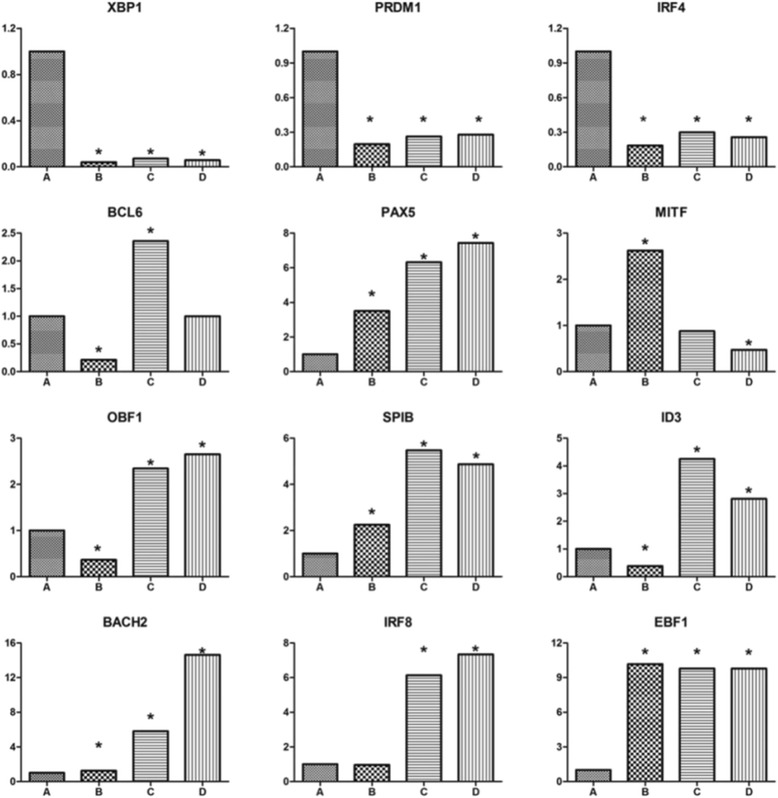
The major transcription factors regulating B-cell differentiation into plasma cells. Analysis of transcription factor expression regulating B-cell differentiation into plasma cells. Bars *A* to *D* indicate CD19^+^CD24^−^CD38^hi^ plasmablasts/plasma cells, regulatory B cells, memory B cells, and naïve B cells, respectively. *Difference of more than twofold by comparison of CD19^+^CD24^−^CD38^hi^ plasmablasts/plasma cells with other B-cell subsets

The transcription factors regulating B-cell differentiation into plasmablasts/plasma cells were analyzed in the various B-cell subsets in greater detail (Fig. 3). In patients with IgG4-RD, B-cell lymphoma 6 protein (*BCL6*) and *PRDM1*, a pair of mutually restrictive transcription factors involved in B-cell differentiation, showed opposite expression in CD19^+^CD24^−^CD38^hi^ plasmablasts/plasma cells compared with the other three B-cell subsets, with *BCL6* expression being lower and *PRDM1* expression higher in CD19^+^CD24^+^CD38^hi^ plasmablasts/plasma cells. In addition, compared with the other three B-cell subsets, the expression of paired box gene 5 (*PAX5*) in CD19^+^CD24^−^CD38^hi^ plasmablasts/plasma cells was much lower. Genes related to *PAX5*, including *IRF8, SPIB, Bach2, EBF, ID3,* and *CIITA*, were also downregulated. These results confirm that CD19^+^CD24^−^CD38^hi^ cells are plasmablasts/plasma cells.

**Fig. 4:**
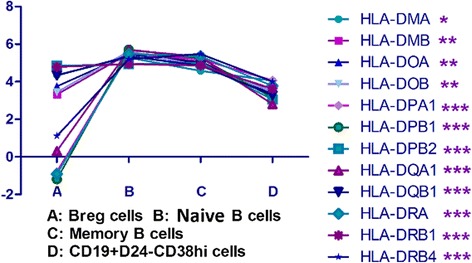
Expression of HLA class II genes in CD19^+^CD24^−^CD38^hi^ plasmablasts/plasma cells, regulatory B cells, memory B cells, and naïve B cells

Next, we analyzed other important genes related to the differentiation of B cells. Compared with the other three B-cell subsets, *CD22* was markedly downregulated in CD19^+^CD24^−^CD38^hi^ plasmablasts/plasma cells, whereas HLA class II genes (*HLA-DRA, HLA-DRB1*), *CIITA*, and *CD19* were significantly downregulated, compared with memory and naïve B cells (Fig. 4, Tables 2 and 3). High expression of *IGHD, IGJ, IGK*, and *IGHM* are important indicators for B-cell differentiation into plasma cells. In CD19^+^CD24^−^CD38^hi^ plasmablasts/plasma cells from patients with IgG4-RD,* IGH* gene expression was also markedly increased, as was that of *IGK* and *IGL* genes. In addition, the expression of *CD27 *and *Fas/CD95* genes was significantly higher than in the other three B-cell subsets, whereas *CD40* gene expression was decreased. *CD23* was also markedly decreased in CD19^+^CD24^−^CD38^hi^ plasmablasts/plasma cells of patients with IgG4-RD.

### Analysis of B-cell proliferation-regulating genes and homing factor genes

**Fig. 5:**
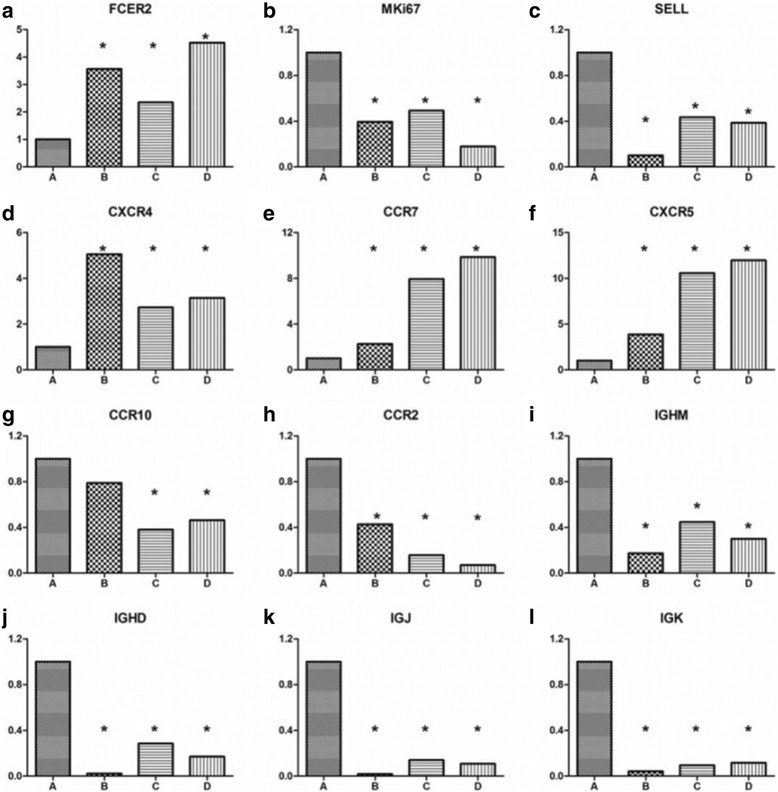
Expression of proliferation genes, homing genes, and immunoglobulin genes in different B-cell subsets. B-cell proliferation genes, homing genes, and immunoglobulin genes were analyzed in B-cell subsets in patients with immunoglobulin G4-related disease. **a** to **l** illustrate genes differently expressed in B cell subsets. Bars *A* to *D* indicate CD19^+^CD24^−^CD38^hi^ plasmablasts/plasma cells, regulatory B cells, memory B cells, and naïve B cells, respectively. *Difference of more than two fold by comparison of CD19^+^CD24^−^CD38^hi^ plasmablasts/plasma cells with other B-cell subsets

Marker of proliferation Ki-67 (*MKI67*), encoding the Ki-67 protein and denoting recent cellular proliferation, was markedly increased in CD19^+^CD24^−^CD38^hi^ plasmablasts/plasma cells compared with the other three B-cell subsets (Fig. 5). We also found that a number of genes involved in cell homing, including *SELL*, which encodes L-selectin (*CD62L*), was markedly increased, whereas expression of *CCR7, CXCR5*, and *CXCR4* was greatly decreased, in CD19^+^CD24^−^CD38^hi^ plasmablasts/plasma cells compared with the other three B-cell subsets. *CCR10* expression was also markedly increased compared with memory B cells and naïve B cells (Fig. 5).

### Expression of activating factors of CD19^+^CD24^−^CD38^hi^ plasmablasts/plasma cells

**Fig. 6:**
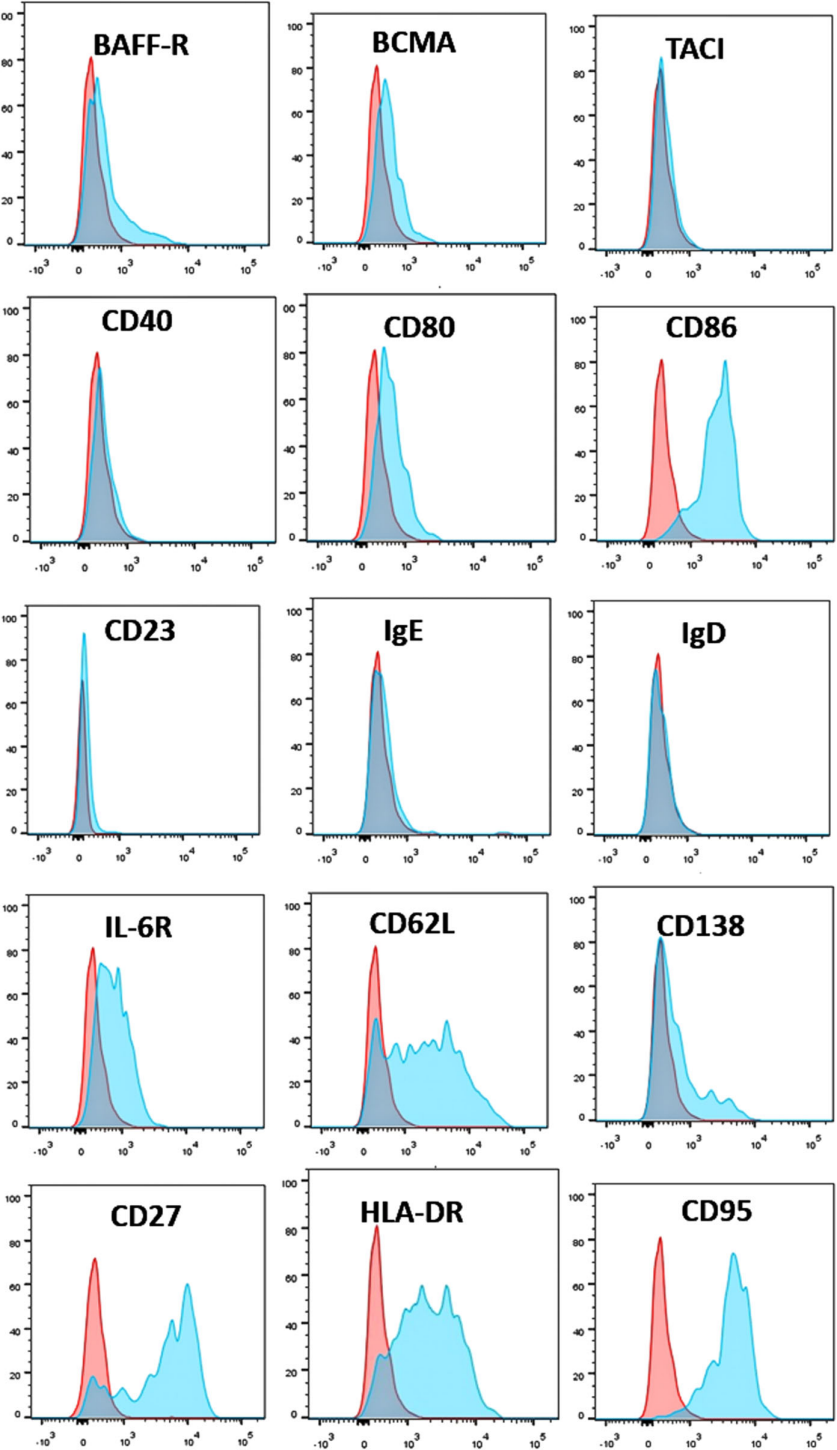
The phenotypic analysis of CD19^+^CD24^−^CD38^hi^ plasmablasts/plasma cells determined by flow cytometry. CD19^+^CD24^−^CD38^hi^ plasmablasts/plasma cells were gated and then analyzed by flow cytometry. *Red* represents isotype control, and *blue* represents the expression level by CD19^+^CD24^−^CD38^hi^ plasmablasts/plasma cells. *BAFF* B-cell activating factor, *BCMA* B-cell maturation antigen, *Ig* Immunoglobulin, *IL-6R* Interleukin-6 receptor, *TACI* Transmembrane activator CAML (calcium modulator and cyclophilin ligand) interactor

By flow cytometric analysis, we compared the expression of B-cell-activating factors and homing factors in different B-cell subsets in patients with IgG4-RD (Fig. 6). This analysis revealed that CD86, CD62L, HLA-DR, and CD95 were highly expressed in CD19^+^C24^−^CD38^hi^ plasmablasts/plasma cells compared with other B-cell subsets. In particular, the majority of CD19^+^CD24^−^CD38^hi^ B cells expressed CD27 (76.46 ± 7.16%), which demonstrated that the CD19^+^CD24^−^CD38^hi^ population was a larger population that included CD19^+^CD20^−^CD27^+^ plasmablasts/plasma cells. Whereas CD138, BAFF-R, and BCMA were expressed by a subset of CD19^+^CD24^−^CD38^hi^ plasma/plasma cells, there was almost no expression of TACI, CD23, CD40, IgE, and IgD by this population.

### Immunoglobulin secretion by different B-cell subsets in IgG4-RD

The CD19^+^C24^−^CD38^hi^ plasmablasts/plasma cells, Bregs, memory B cells, and naïve B cells from patients with IgG4-RD were isolated by cell sorting and cultured in vitro. At day 7, the supernatants were collected, and the secretion of immunoglobulin was tested.

**Fig. 7:**
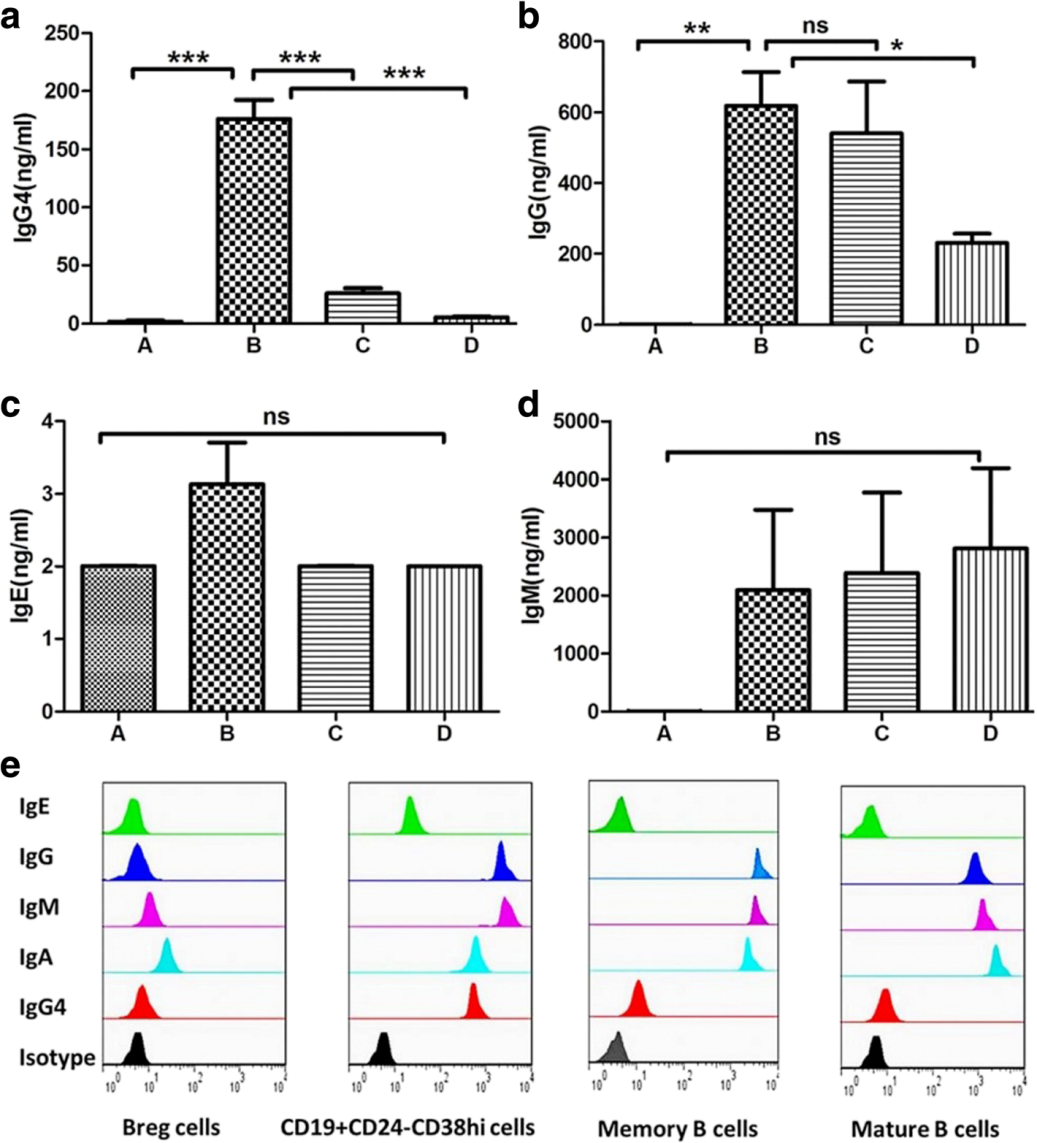
The secretion of immunoglobulin by regulatory B cells (Bregs), CD19^+^C24^−^CD38^hi^ plasmablasts/plasma cells, memory B cells, and naïve B cells. B-cell subsets, including CD19^+^CD24^−^CD38^hi^ plasmablasts/plasma cells, Bregs, memory B cells, and naïve B cells from peripheral blood of patients with immunoglobulin G4-related disease (IgG4-RD) were sorted by flow cytometry and underwent cell culture in vitro. After 7 days of cell culture with CD40L and CpG ODN 2006, the supernatants were collected, and immunoglobulin secretion was analyzed. *Horizontal histogram* illustrates (**a**) IgG4, (**b**) IgG, (**c**) IgE, and (**d**) IgM secreted by four subsets of B cells. Columns *A*–*D* represent Bregs, CD19^+^C24^−^CD38^hi^ plasmablasts/plasma cells, memory B cells, and naïve B cells, respectively. **e** Flow diagram of a representative patient with IgG4-RD. (*: *P* <0.05, **: *P* <0.01, ***: *P* <0.001)

The results showed that the IgG secreted by CD19^+^CD24^−^CD38^hi^ plasmablasts/plasma cells was 618.4 ± 163.3 ng/ml, much higher than that of Bregs and naïve B cells (0.72 ± 0.59 ng/ml and 231.2 ± 44.83 ng/ml, respectively; (*P* = 0.003 and *P* = 0.02, respectively), but that it was not different from that of memory B cells (541.0 ± 252.1 ng/ml, *P* = 0.678). In addition, the IgG4 secreted by CD19^+^C24^−^CD38^hi^ plasmablasts/plasma cells was 176.1 ± 28.5 ng/ml, significantly higher than that secreted by Bregs, memory B cells, and naïve B cells (0.87 ± 0.59 ng/ml, *P* = 0.0005; 26.24 ± 7.08 ng/ml, *P* = 0.0009; and 5.34 ± 1.50 ng/ml, *P* = 0.0005, respectively). CD19^+^CD24^−^CD38^hi^ plasmablasts/plasma cells also secreted more IgE than other B-cell subsets (3.14 ± 0.99 ng/ml, 2.01 ± 0.01 ng/ml, 2.01 ± 0.01 ng/ml, and 2.00 ± 0.01 ng/ml, respectively; *P* = 0.054). Regarding other immunoglobulins, there was no significant difference in secreted IgA (20,573 ± 22,215 ng/ml, 12.39 ± 1.96 ng/ml, 5209 ± 1066 ng/ml, and 12105 ± 16970 ng/ml, respectively; *P* = 0.37) and secreted IgM (2090 ± 2401 ng/ml, 8.34 ± 1.75 ng/ml, 2385 ± 2402 ng/ml, and 2810 ± 2387 ng/ml, respectively; *P* = 0.41) compared with CD19^+^C24^−^CD38^hi^ plasmablasts/plasma cells and the other three subsets (Fig. 7).

### Correlations of CD19^+^CD24^−^CD38^hi^ plasmablasts/plasma cells with disease activity and changes with treatment

**Fig. 8:**
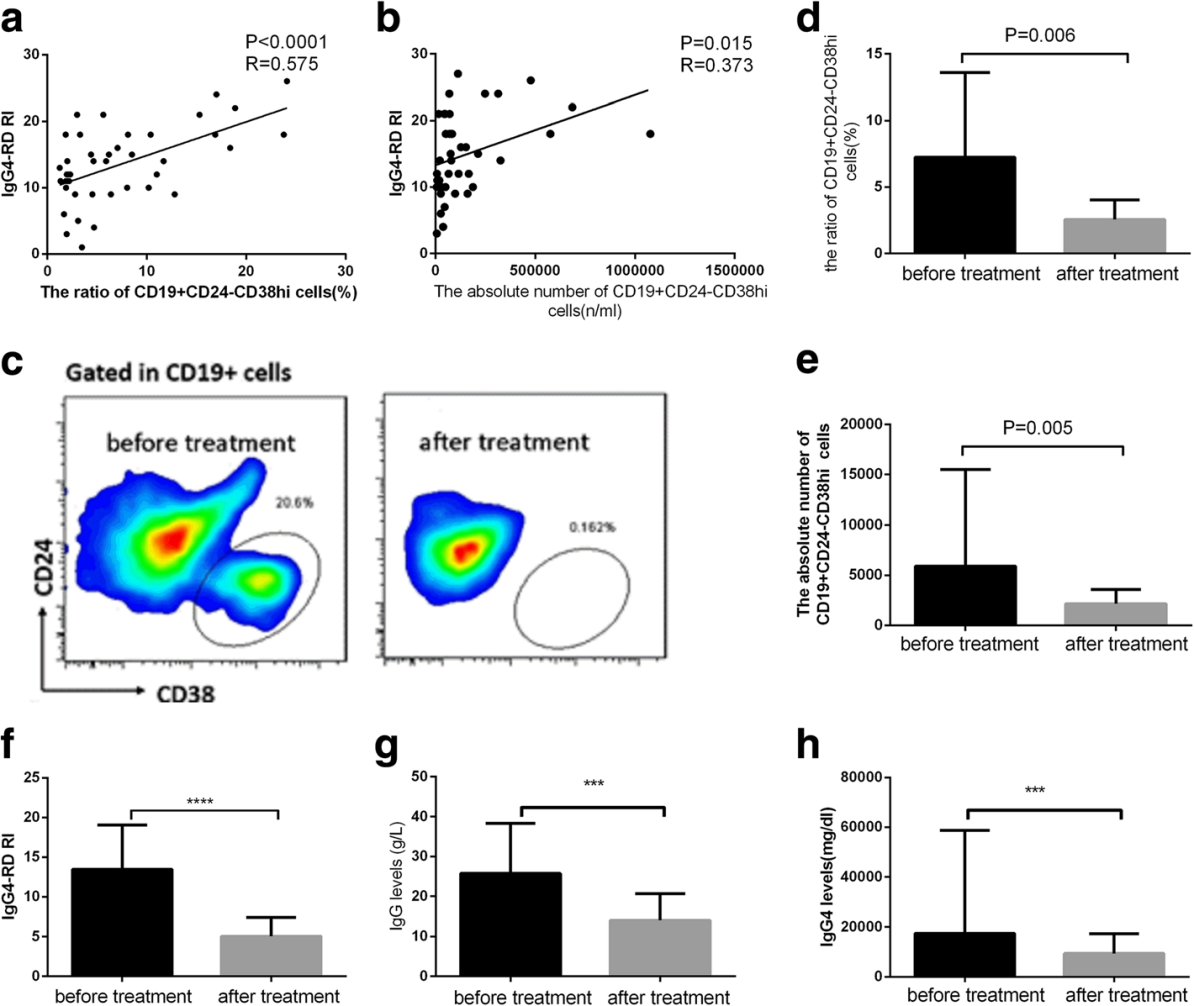
The association between CD19^+^CD24^−^CD38^hi^ plasmablasts/plasma cells and disease activity. **a** and **b** Correlations of the ratio of CD19^+^CD24^−^CD38^hi^ cells/CD19^+^ B cells and the absolute number of CD19^+^CD24^−^CD38^hi^ plasmablasts/plasma cells with IgG4-related Disease Responder Index (IgG4-RD RI) before treatment. **c** Change of CD19^+^CD24^−^CD38^hi^ plasmablasts/plasma cells before and after treatment. **d**–**h** Ratios of CD19^+^CD24^−^CD38^hi^ cells/CD19^+^ B cells, absolute number of CD19^+^CD24^−^CD38^hi^ plasmablasts/plasma cells, IgG4-RD RI, IgG, and IgG4 before and after treatment. *** *P* < 0.001, **** *P* < 0.0001

Forty-two patients with IgG4-RD were assessed before treatment with glucocorticoids, and 15 patients with IgG4-RD were assessed both before and after treatment with glucocorticoids. Before treatment, both the ratio of CD19^+^CD24^−^CD38^hi^ cells/CD19^+^ B cells and the absolute number of CD19^+^CD24^−^CD38^hi^ cells correlated positively with disease activity in terms of IgG4-RD RI (*R* = 0.575, *P* < 0.0001; *R* = 0.373, *P* = 0.015, respectively) (Fig. 8a, b). After treatment, along with the decrease of IgG4-RD RI (from 13.48 ± 5.60 to 5.06 ± 2.38, *P* < 0.009) (Fig. 8f), serum IgG (from 25.74 g/L ± 12.55 g/L to 14.06 g/L ± 6.23 g/L, *P* < 0.001), and IgG4 (from 1740 mg/dl [7160–58,700] to 938 mg/dl [2900–17,300], *P* < 0.0001) (Fig. 8g, h), both the absolute number (from 5914 [1888–19,487]/ml to 2157 [822–3582]/ml, *P* = 0.005) and the ratio (from 7.25% ± 6.35% to 2.55% ± 1.50%, *P* < 0.001) of CD19^+^C24^−^CD38^hi^ plasmablasts/plasma cells decreased significantly (Fig. 8c–e). By contrast, circulating memory B cells, which were also increased in untreated patients [17], did not show significant changes after steroid treatment. Notably, however, there was no significant correlation between the magnitude of decrease of the ratio of CD19^+^CD24^−^CD38^hi^ plasma cells and the decrease of the IgG4-RD RI (*R* = 0.505, *P* = 0.055) (data not shown).

## Discussion

The data derived from the present study indicate that CD19^+^CD24^−^CD38^hi^ plasmablasts/plasma cells are significantly elevated in the peripheral blood of patients with IgG4-RD, and this B-cell population notably correlates with the number of organs involved, serum IgG4, IgG4-RD RI, and the ratio of IgG4/IgG. In addition, peripheral CD19^+^CD24^−^CD38^hi^ plasmablasts/plasma cells decreased remarkably after treatment of glucocorticoids, and the clinical manifestations of patients improved accordingly. These findings are consistent with previous observations [17] and indicate that the level of CD19^+^CD24^−^CD38^hi^ plasmablasts/plasma cells could be an important biomarker in IgG4-RD.

By gene expression analysis, we found that CD19^+^CD24^−^CD38^hi^ plasmablasts/plasma cells differed greatly from the other three subsets of B cells, including naïve B cells, memory B cells, and Bregs, and expressed many genes typical of plasma cells. Considering that the gene expression of CD19^+^CD24^−^CD38^hi^ plasmablasts/plasma cells differed from that of other known B-cell subsets, we attempted to use gene expression analysis to determine their stage of plasma cell maturation. A number of typical plasma cell genes were overexpressed, including XBP1, PRDM1, IRF4, and TNFRSF17, whereas BCL6, SPIB, and PAX5 were underexpressed, which is typical of mature plasma cells. However, expression of these genes does not permit a more detailed analysis of the stage of maturation of plasma cells. It is known, however, that expression of HLA-DR can be employed to identify newly generated cells from more mature plasma cells; in this regard, CD19^+^CD24^−^CD38^hi^ plasmablasts/plasma cells from patients with IgG4-RD exhibited bright expression of HLA-DR protein, as is typical of newly formed plasma cells [27]. Notably, however, messenger RNA (mRNA) levels of various major histocompatibility complex class II molecules were decreased, as has been noted in systemic lupus erythematosus (SLE) [22], suggesting that these plasma cells were transitioning from newly formed to more mature plasma cells that had decreased mRNA expression but retained protein expression of HLA-DR. Consistent with this, IgG4-RD plasma cells greatly overexpressed Mki-67 mRNA, which encodes the Ki-67 protein, a marker of recently divided immunoglobulin-secreting plasma cells [28] and which is increased in plasmablasts compared with memory B cells [29]. Together, the increased expression of HLA-DR protein but not mRNA and the increase in Mki-67 mRNA identify IgG4-RD plasma cells as newly generated and similar to those found in the circulation of patients with SLE [30].

A number of other mRNAs and proteins were assessed to further understand the maturation status of the IgG4-RD plasma cells. The presence of mRNA for both lambda and kappa light chains suggests that these CD19^+^CD24^−^CD38^hi^ plasmablasts/plasma cells are polyclonal, although more detailed analysis on a single-cell level would be required to confirm this. An interesting feature of CD19^+^CD24^−^CD38^hi^ plasmablasts/plasma cells was their decreased expression of CXCR4. This chemokine receptor uniquely recognizes CXCL12 and plays an important role in plasma cell homing to bone marrow and other niches [31, 32]. The decreased expression of CXCR4 could contribute to their persistence in the circulation. A similar abnormality has been noted in SLE plasma cells [22].

To further identify the characteristics of CD19^+^CD24^−^CD38^hi^ plasmablasts/plasma cells, we analyzed the surface markers by flow cytometry, and we found that CD19^+^CD24^−^CD38^hi^ plasmablasts/plasma cells highly expressed CD86, CD62L, IL-6R, CD27, and CD95, which was consistent with those observed in gene levels. CD86 (B7-2) signaling plays a pivotal role in activating T cells [33]. Expression of CD86 on B-lineage cells has been shown to foster B-cell–T-cell collaboration and facilitate immunoglobulin production, including IgE and IgG4 [34–39]. In IgG4-RD, the CD19^+^CD24^−^CD38^hi^ plasmablasts/plasma cell subset highly expressed the CD86 molecule, suggesting the intriguing possibility that this cell subset may maintain some features of earlier B cells and, by virtue of persistent expression of CD86, chronically stimulate T-cell help and in so doing enhance the likelihood of class-switching recombination to the downstream heavy-chain isotypes IgG4 and IgE. The physiologic role of other molecules expressed by CD19^+^CD24^−^CD38^hi^ plasmablasts/plasma cells requires delineation.

As previously reported, circulating plasmablasts identified using CD19^low^CD38^+^CD20^−^CD27^+^ phenotypic markers are significantly elevated in active IgG4-RD, even in patients with normal serum IgG4 concentrations, suggesting that plasmablast counts are a potentially useful biomarker for diagnosis of IgG4-RD as well as for assessing response to treatment [13]. In our study, we found that 76.46% of CD19^+^CD24^−^CD38^hi^ cells expressed CD27, indicating that the CD19^+^CD24^−^CD38^hi^ plasmablasts/plasma cell population is a larger one that contained CD19^+^CD20^−^CD27^+^CD38^hi^ cells. Similarly, the ratio of CD19^+^CD24^−^CD38^hi^ cells correlated positively with IgG4-RD RI, as did the absolute number of CD19^+^CD24^−^CD38^hi^ plasmablasts/plasma cells [17]. Although there was no statistical correlation between the magnitude of change in circulating CD19^+^CD24^−^CD38^hi^ plasmablasts/plasma cells and the change in the IgG4-RD RI, this may emerge when more patients are studied. Moreover, the significant correlation between disease activity and circulating plasmablasts/plasma cells, as well as the decrease in circulating plasma cells with therapy, suggests that this might be useful in assessing these patients and their response to treatment.

## Conclusions

In this study, we found that CD19^+^CD24^−^CD38^hi^ plasmablasts/plasma cells are prominently increased in peripheral blood of untreated patients with IgG4-RD, correlating positively with serum IgG4, IgG4/IgG ratio, IgG4-RD RI, and the number of involved organs. This population decreased significantly after steroid treatment, suggesting that CD19^+^CD24^−^CD38^hi^ plasmablasts/plasma cells may be a biomarker of IgG4-RD and potentially could be useful in confirming a diagnosis, monitoring response to therapy, or assessing disease activity.

## Abbreviations

ANOVA: Analysis of variance
BAFF: B-cell activating factor
BAFF-R: B-cell activating factor receptor
BCL-6: B-cell lymphoma 6 protein
BCMA: B-cell maturation antigen
BCR: B-cell receptor
Breg: Regulatory B cell
CBA: Cytometric bead array
cDNA: Complementary DNA
CRP: C-reactive protein
Cy: Cyanine
ESR: Erythrocyte sedimentation rate
HLA: Human leukocyte antigen
Ig: Immunoglobulin
IgG4: Immunoglobulin G4
IgG4-RD: Immunoglobulin G4-related disease
IgG4-RD RI: IgG4-related Disease Responder Index
IL: Interleukin
IRF4: Interferon regulatory factor 4
mAb: Monoclonal antibody
MKI67: Marker of proliferation Ki-67
mRNA: Messenger RNA
PAX5: Paired box gene 5
PBMC: Peripheral blood mononuclear cell
PE: Phycoerythrin
SLE: Systemic lupus erythematosus
SPIB: Transcription factor Spi-B
SSC: Side scatter
TACI: Transmembrane activator CAML (calcium modulator and cyclophilin ligand) interactor
TNFRSF: Tumor necrosis factor receptor superfamily member 17
XBP1: X-box binding protein 1
